# Obese cardiogenic arrest survivors with significant coronary artery disease had worse in-hospital mortality and neurological outcomes

**DOI:** 10.1038/s41598-020-75752-9

**Published:** 2020-10-29

**Authors:** Chih-Wei Sung, Chien-Hua Huang, Wen-Jone Chen, Wei-Tien Chang, Chih-Hung Wang, Yen-Wen Wu, Wei-Ting Chen, Jia-How Chang, Min-Shan Tsai

**Affiliations:** 1 Department of Emergency Medicine, National Taiwan University Hospital Hsin-Chu Branch, Hsinchu City, Taiwan; 2 Department of Emergency Medicine, National Taiwan University Medical College and Hospital, Taipei City, Taiwan; 3 Division of Cardiology, Department of Internal Medicine, National Taiwan University Hospital, Taipei City, Taiwan; 4 Department of Nuclear Medicine, Cardiology Division of Cardiovascular Medical Center, Far Eastern Memorial Hospital, New Taipei City, Taiwan

**Keywords:** Outcomes research, Myocardial infarction

## Abstract

Cardiogenic arrest is the major cause of sudden cardiac arrest (SCA), accounting for 20% of all deaths annually. The association between obesity and outcomes in cardiac arrest survivors is debatable. However, the effect of obesity on the prognosis of patients with significant coronary artery disease (CAD) successfully resuscitated from cardiogenic arrest is unclear. Thus, the association between body mass index (BMI) and outcomes in cardiogenic arrest survivors with significant CAD was investigated. This multicentre retrospective cohort study recruited 201 patients from January 2011 to September 2017. The eligible cardiogenic arrest survivors were non-traumatic adults who had undergone emergency coronary angiography after sustained return of spontaneous circulation and had significant coronary artery stenosis. BMI was used to classify the patients into underweight, normal-weight, overweight, and obese groups (< 18.5, 18.5–24.9, 25.0–29.9, and ≥ 30 kg/m^2^; n = 9, 87, 72, and 33, respectively). In-hospital mortality and unsatisfactory neurological outcomes (cerebral performance scale scores = 3–5) were compared among the groups. The obese group presented higher in-hospital mortality and unsatisfactory neurological outcome risks than the normal-weight group (in-hospital mortality: adjusted hazard ratio = 4.27, 95% confidence interval (CI) 1.87–12.04, P = 0.008; unsatisfactory neurological outcomes: adjusted odds ratio = 3.33, 95% CI 1.42–8.78, P = 0.009). Subgroup analysis showed significantly higher in-hospital mortality in the obese patients than in the others in each clinical characteristic. In cardiogenic arrest survivors with significant CAD, obesity was associated with high risks of mortality and unsatisfactory neurological recovery.

## Introduction

Obesity, with its increasing prevalence, is becoming a crucial public health concern in recent years. In 2011, the World Health Organization (WHO) reported a significant increase in the prevalence of obesity in the United States; 62% of the adult population was categorised as overweight, including 26% obese adults^1^. Obesity is correlated with several comorbidities, such as metabolic syndrome, coronary artery disease (CAD), neoplasm, musculoskeletal disorders, and idiopathic sudden cardiac arrest (SCA)^2,3^. Obese patients account for 300,000 SCA cases in the United States annually^3^. Therefore, the association between obesity and outcomes in patients experiencing cardiac arrest is receiving increasing scientific attention.

Cardiogenic arrest, the major cause of SCA, contributes to 20% of all deaths annually^4^. Because patients with cardiogenic arrest are relatively young, mortality and unsatisfactory prognosis among these patients cause a considerable economic and social burden^5^. Cardiogenic arrest may be caused by CAD, acute myocardial infarction, heart failure, cardiac tamponade, or fatal arrhythmia^5,6^, which have been reported to be attributed to obesity. Obesity was also reported to increase the risk of sudden arrhythmic death syndrome and left ventricular hypertrophy^7^. Compared with the normal-weight population, obese men and women have a 2.6- and 5.8-fold higher risk of SCA, respectively^8^.

Resuscitating obese patients is more challenging than resuscitating normal-weight patients because of difficulties in providing adequate chest compressions, ventilation, and oxygenation^9^. Obese patients usually require cardiopulmonary resuscitation (CPR) for a longer duration, which increases the cerebral ischaemic time, thereby deteriorating neurological outcomes. Obesity indices, such as body mass index (BMI), were reported to act as independent makers in 30-day mortality^10^. However, an obesity paradox was noted. The overall survival condition and neurological prognosis of overweight or obese patients were superior to those of normal-weight patients because overweight or obese patients may have greater metabolic reserves in critical life-threatening scenarios^9,11–13^. However, two recent meta-analyses have shown that survival rates and neurological outcomes did not differ significantly between obese and non-obese patients^6,14^.

Our previous study demonstrated that the presumed cardiogenic arrest survivors with an obese BMI had higher risk for in-hospital mortality and poor neurological recovery^15^. However, the association between obesity and outcomes in cardiogenic arrest survivors with significant coronary artery stenosis after return of spontaneous circulation (ROSC) remains unclear. We conducted a retrospective cohort study to investigate the association between obesity and outcomes in patients who had significant coronary artery stenosis and were successfully resuscitated using emergency coronary angiography (CAG).

## Methods

### Study design, setting, and population

A multicenter retrospective cohort study was conducted in National Taiwan University Hospital (NTUH), NTUH Hsin-Chu Branch (HCH), and Far Eastern Memorial Hospital (FEMH), which are tertiary-care medical centers in Taiwan. The study was approved by the institutional review boards of NTUH, HCH and FEMH. The requirement of informed consent was waived and it also approved by the institutional review boards of the aforementioned hospitals. All research was performed in accordance with relevant guidelines or regulations.

**Figure 1:**
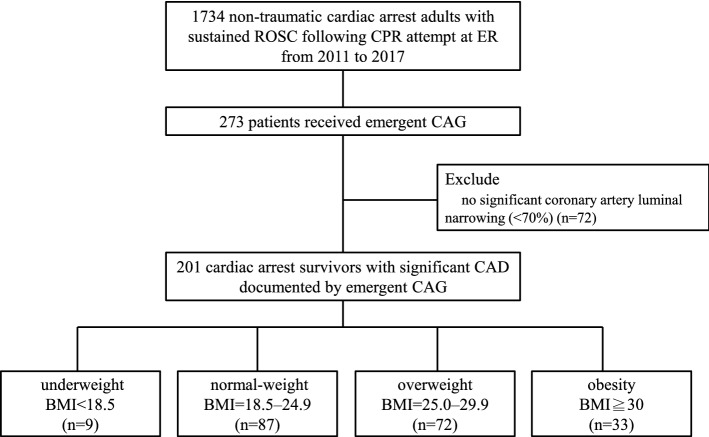
Flowchart of patient enrolment.

The patients were recruited from NTUH between January 2011 and July 2017, from HCH between 2015 and 2017, and from FEMH between 2011 and 2015. A total of 1734 non-traumatic adult cardiac arrest survivors with sustained ROSC in the emergency room after CPR were initially recruited. Among them, 273 patients received emergency CAG within 24 h of ROSC. The patients without significant coronary artery stenosis, defined as less than 70% stenosis in a major coronary artery, detected using emergency CAG were excluded. A total of 201 cardiac arrest survivors were enrolled in the final analysis with significant CAD that was defined as any coronary artery stenosis in index emergency CAG. The enrolled patients were then divided into four groups based on the WHO classification of BMI^16^, namely underweight (≤ 18.5 kg/m^2^; n = 13, 4.5%), normal-weight (18.5–24.9 kg/m^2^; n = 121, 43.3%), overweight (25.0–29.9 kg/m^2^; n = 100, 35.8%), and obese (≥ 30.0 kg/m^2^; n = 39, 16.4%) (Fig. 1).

### Data collection and variables

Data of the patients were collected from medical records by using a predefined data collection form. The following variables were collected: age, sex, BMI, comorbidities, resuscitation events (witnessed collapse, bystander CPR, initial shockable rhythm, and CPR duration during the resuscitation period), and post-arrest management [emergency percutaneous coronary intervention (PCI), the use of an inotrope, therapeutic hypothermia, extracorporeal membrane oxygenation (ECMO), intra-aortic balloon pump (IABP), and dual antiplatelet agents (DAPT)]. The target temperature of therapeutic hypothermia was kept the body temperature at 33 °C for 24 h in post-cardiac arrest patients. The administration of inotropes and emergency PCI, ECMO, IABP, and DAPT were considered when these therapies were applied within 7 days of ROSC. Body weight at index intensive care unit (ICU) admission of each patient was recorded using a standard electronic body weightometer; BMI was subsequently calculated.

### Outcomes

The primary outcome of this study was in-hospital mortality and the secondary outcome is neurological recovery at hospital discharge. The follow-up period was from index admission to two months after hospitalization. Neurological function was assessed clinically by attending physicians by using the Glasgow-Pittsburgh Cerebral Performance Category (CPC) scores, in which CPC 1 or 2 indicated satisfactory neurological function while CPC 3–5 indicated unsatisfactory neurological function^17^.

### Statistical analysis

Continuous variables with normal distribution were presented as mean ± standard deviation and were compared between groups by using analysis of variance. Categorical variables were presented as numbers (percentages) and were compared between the groups by using a chi-squared test (χ^2^-test). A Kruskal–Wallis analysis was performed to test for the normality of the variables. The effects of BMI on in-hospital mortality and neurological outcomes were determined using a Cox proportional hazards model and multiple logistic regression analysis, respectively. The variables initially identified with P < 0.1 in the univariate analysis were subsequently included in Cox proportional hazards models and multiple logistic regression analysis for final analysis. Hazard ratios (HRs) and 95% confidence intervals (CIs) were determined in the Cox proportional hazards models. Odds ratios (ORs) and 95% CIs were determined in multiple logistic regression analysis. The cumulative risk of in-hospital mortality was illustrated using the cumulative incidence function and compared by using the log-rank test. The interactions between obesity and each significant variable for in-hospital mortality were presented using a forest plot. SAS (version 9.4, Chicago, IL) was used for all statistical analyses; the significance level was set at P ≤ 0.05.

## Results

Table 1 presents the baseline characteristics, CPR events, and post-arrest care of the all the patients together as well as after classification into groups. The patients with higher BMI were younger in those with lower BMI (underweight patient age: 69.87 ± 9.25 years, normal-weight patient age: 63.88 ± 11.32 years, overweight patient age: 61.87 ± 11.39 years, and obese patient age: 57.19 ± 14.28 years; P = 0.011). The CPR duration significantly increased with an increase in BMI (obese patients: 28.5 ± 35.0 min, overweight patients: 27.5 ± 36.0 min, normal-weight patients: 13.0 ± 22.0 min, and underweight patients: 18.0 ± 17.0 min, P = 0.041). The obese group exhibited increased inotrope and ECMO use following ROSC. There were 143 patients (71.1%) receiving emergent PCI and 58 patients (28.9%) receiving CAG only. No significant difference was noted between BMI groups.

**Table 1:** Baseline characteristics between groups with BMI.

	BMI					*p*
	Total patients	Underweight < 18.5	Normal-weight 18.5–24.9	Overweight 25.0–29.9	Obesity ≧ 30	
	n = 201	n = 9 (4.48%)	n = 87 (43.28%)	n = 72 (35.82%)	n = 33 (16.42%)	
Age (years)	61.84 ± 12.74	69.87 ± 9.25	63.88 ± 11.32	61.87 ± 11.39	57.19 ± 14.28	0.011
Gender (Male)	167 (83.08%)	8 (88.89%)	71 (81.61%)	61 (84.72%)	27 (81.82%)	0.916
Smoking	47 (23.38%)	2 (22.22%)	20 (22.99%)	16 (22.22%)	9 (27.27%)	0.926
*Comorbidities*						
Hypertension	119 (59.20%)	4 (44.44%)	47 (54.02%)	47 (65.28%)	21 (63.64%)	0.331
Diabetes mellitus	85 (39.56%)	1 (11.11%)	35 (40.23%)	32 (44.44%)	17 (51.52%)	0.145
Dyslipidemia	44 (21.89%)	3 (33.33%)	25 (28.74%)	13 (18.06%)	3 (9.09%)	0.086
Coronary artery disease	83 (41.29%)	4 (44.44%)	33 (37.93%)	31 (43.06%)	15 (45.45%)	0.816
Previous stent deployment	0.41 ± 0.92	0.11 ± 0.33	0.43 ± 0.92	0.58 ± 1.23	0.68 ± 1.23	0.422
Myocardial infarction	7 (3.48%)	0 (0.00%)	4 (4.59%)	3 (4.17%)	0 (0.00%)	0.595
Cardiac arrhythmia	12 (5.97%)	0 (0.00%)	6 (6.89%)	4 (5.56%)	2 (6.06%)	0.867
Heart failure	15 (7.46%)	0 (0.00%)	5 (5.75%)	9 (12.50%)	1 (3.03%)	0.204
Chronic kidney disease	38 (18.91%)	0 (0.00%)	18 (20.69%)	14 (19.44%)	6 (18.18%)	0.516
Malignancy	9 (4.76%)	0 (0.00%)	5 (5.75%)	4 (5.56%)	0 (0.00%)	0.487
*Resuscitation events*						
Witnessed collapse	161 (80.10%)	7 (76.92%)	74 (85.06%)	58 (80.56%)	22 (66.67%)	0.197
Bystander CPR	143 (73.43%)	5 (55.56%)	65 (74.71%)	52 (72.22%)	21 (63.64%)	0.482
Initial shockable rhythm	79 (39.30%)	2 (23.08%)	37 (42.53%)	29 (40.28%)	11 (33.33%)	0.372
Non-shockable rhythm	81 (40.30%)	3 (33.33%)	33 (37.93%)	30 (41.67%)	15 (45.46%)	0.871
CPR duration (mins)	20 ± 22	18 ± 17	13 ± 22	27.5 ± 36	28.5 ± 35	0.041
*Post-ROSC events*						
Inotrope use	154 (76.62%)	5 (55.56%)	64 (73.56%)	55 (76.39%)	30 (90.91%)	0.053
Therapeutic hypothermia	69 (34.33%)	2 (23.08%)	34 (39.08%)	22 (30.56%)	11 (33.33%)	0.489
ECMO	85 (42.29%)	3 (33.33%)	32 (36.78%)	30 (41.67%)	20 (60.61%)	0.081
Intra-aortic balloon pump	90 (44.78%)	3 (33.33%)	39 (44.83%)	28 (38.89%)	20 (60.61%)	0.137
Emergent PCI	143 (71.14%)	5 (55.56%)	64 (73.56%)	50 (69.44%)	24 (72.73%)	0.649
Dual anti-platelet agents	161 (80.10%)	7 (76.92%)	70 (80.46%)	59 (81.94%)	25 (75.76%)	0.964
CPC scores ≤ 2	90 (44.78%)	4 (44.44%)	46 (52.87%)	32 (44.44%)	8 (24.24%)	0.006

*BMI* body mass index, *CPC* cerebral performance category, *CPR* cardiopulmonary resuscitation, *ECMO* extracorporeal membrane oxygenation, *FEMH* Far Eastern Memorial Hospital, *NTUH* National Taiwan University Hospital, *HCH* National Taiwan University Hospital Hsin-Chu branch, *PCI* percutaneous coronary intervention, *ROSC* return of spontaneous circulation.

*p < 0.05.

**Figure 2:**
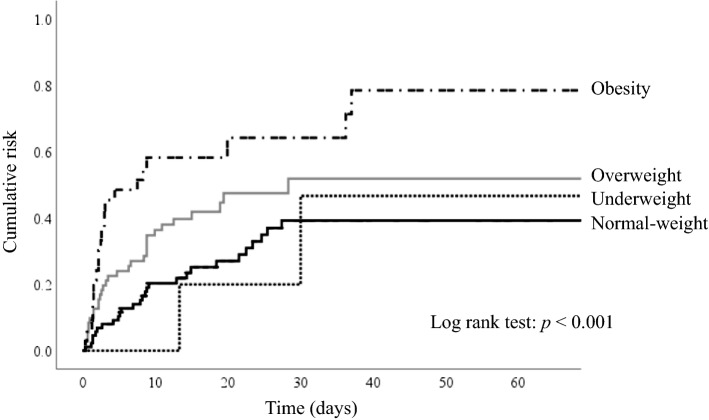
Cumulative risk for in-hospital mortality between groups log-rank test *p* < 0.001.

In total, 24 (72.7%), 4 (44.4%), 31 (35.6%), and 34 (47.2%) patients in the obese, underweight, normal-weight, and overweight groups, respectively, did not survive until hospital discharge. The confounders were adjusted including age, gender, diabetes mellitus, hypertension, coronary artery disease, witnessed collapse, bystander CPR, CPR duration, post-arrest inotrope use, ECMO, IABP, vessel number of coronary artery stenosis, vessel number of non-revascularized coronary artery, emergent PCI after ROSC. The risk of in-hospital mortality in the obese group was significantly higher than that in the normal-weight group [adjusted HR (aHR) = 4.27, 95% CI = 1.87–12.04, P = 0.008] (Table 2). In the obese, underweight, normal-weight, and overweight groups, 25 (75.8%), 5 (55.6%), 41 (47.1%), and 40 (55.6%) patients, respectively, exhibited unsatisfactory neurological recovery. The obese group exhibited a significantly higher risk of unsatisfactory neurological recovery than did the normal-weight group [adjusted OR (aOR) = 3.33, 95% CI = 1.42–8.78, P = 0.009] (Table 2). Figure 2 illustrates the cumulative risk of in-hospital mortality among the groups. The obese group exhibited a higher cumulative risk for in-hospital mortality (log-rank test: P < 0.001) than the other groups.

**Table 2:** Outcomes between different BMI groups.

	In-hospital mortality	Unadjusted HR (95% CI)	*p*	Adjusted HR* (95% CI)	*p*
Underweight	4/9 (44.44%)	1.45 (0.36, 4.78)	0.603	1.38 (0.41–4.97)	0.612
Normal-weight	31/87 (35.63%)	Reference		Reference	
Overweight	34/72 (47.22%)	1.62 (0.85, 3.06)	0.141	1.59 (0.84–3.11)	0.137
Obesity	24/33 (72.73%)	4.62 (1.90–11.21)	0.007	4.27 (1.87–12.04)	0.008

	Poor neurological outcome	Unadjusted OR (95% CI)	*p*	Adjusted OR* (95% CI)	*p*
Underweight	5/9 (55.56%)	1.40 (0.35, 5.58)	0.632	1.38 (0.36, 5.12)	0.624
Normal-weight	41/87 (47.13%)	Reference		Reference	
Overweight	40/72 (55.56%)	1.40 (0.75, 2.63)	0.291	1.23 (0.79, 2.45)	0.301
Obesity	25/33 (75.76%)	3.37 (1.36, 8.31)	0.009	3.33 (1.42, 8.78)	0.009

*BMI* body mass index, *HR* hazard ratio, *OR* odds ratio.

*Adjusted for age, gender, diabetes mellitus, hypertension, coronary artery disease, witnessed collapse, bystander CPR, CPR duration, post-arrest inotrope use, ECMO, IABP, coronary artery stenosis, non-revascularized coronary artery, emergent PCI after ROSC.

**Figure 3:**
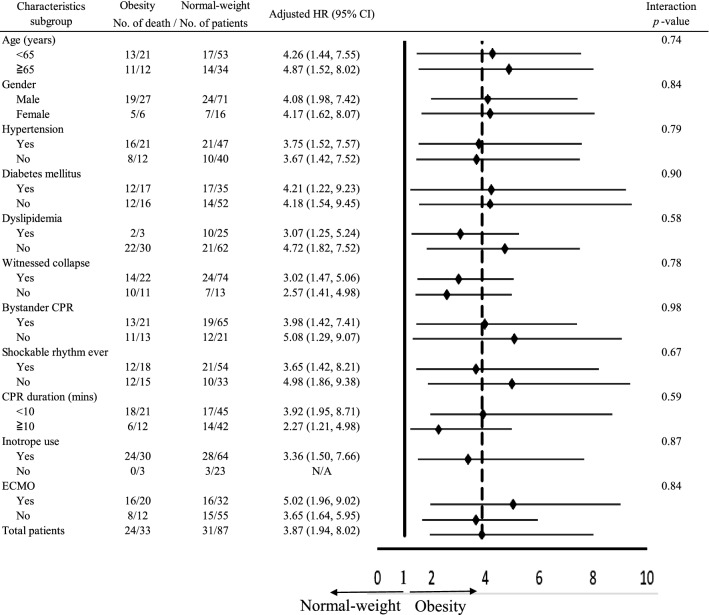
Subgroup analysis of in-hospital mortality interacted with individual clinical characteristics.

The obese group consistently had a significantly higher risk of in-hospital mortality regardless of age, sex, pre-existing diseases (hypertension, DM, and dyslipidaemia), witnessed collapse, bystander CPR, CPR duration, or post-resuscitation care (Fig. 3).

The majority of involved coronary arteries was left anterior descending artery, ranged from 66.7 to 87.9% between groups. The right coronary artery was the second involved coronary artery which was from 44.4 to 69.7%. However, no significant differences were found between BMI groups in all coronary arteries. After PCI, the TIMI grade flow in most involved coronary artery were TIMI 2 or TIMI 3 (Supplement Table 1).

## Discussion

The current study investigated the association between BMI and outcomes in cardiogenic arrest survivors with significant CAD and demonstrated that the obese patients had higher risks of in-hospital mortality and unsatisfactory neurological recovery than the normal-weight patients. With the alternation of daily habit including high calorie diet and insufficient daily activity, the obesity changes the etiology and outcomes in many diseases. In spite of well-known cause-effect relationship between obesity and many diseases, the importance obesity on cardiovascular disease is worthy of being emphasized without doubt. To the best of our knowledge, this study is the first to examine the effects of obesity on prognosis in cardiogenic arrest survivors with significant CAD documented using emergency CAG within 24 h. This study also provides and extends the evidence of obesity on cardiogenic arrest survivors.

The relationship between BMI and all-cause mortality is generally a U-shaped curve with an increased mortality at both boundary levels^18^. A similar U-shaped association was observed between CPR duration and BMI in the present study. The lean patients required CPR for relatively longer durations and experienced difficulty in achieving sustained ROSC. This observation may be explained by low metabolic reserves, unsatisfactory immune responses, and low ability to respond to an acute illness in the lean patients^19^. The patients with high BMI also required CPR for longer durations than did the patients in the normal-weight group^20^ because of difficulties in airway management, impaired ventilation, and long transportation during resuscitation^21,22^. Therefore, at both low and high BMI values, a longer CPR duration prolongs the brain ischaemic time, which deteriorates outcomes eventually.

With an increase in income and quality of life, the rate of obesity in the middle-aged population is higher than that in the elderly population (age > 65 years)^23^. Although the obese patients are young, they have multiple the obesity-related comorbidities such as metabolic syndrome and CAD^2^. The adverse effects of obesity on cardiovascular haemodynamics, structure, impaired systolic and/or diastolic ventricular function, and increased risk of cardiac failure have been widely reported^24,25^. Increased obesity-related cardiac output, metabolic dysregulation, myocardial fatty infiltration all contribute to the eccentric and concentric hypertrophy characteristic of obesity-related cardiac failure^26^, which is also a cause of cardiogenic arrest^27^. Our results demonstrated a quasi-higher percentage of inotrope use in the overweight (76%) and obese patients (91%), as well as higher percentage of ECMO use (60%) in the obese patients. The results may imply more critical cardiovascular status after ROSC in the obese patients.

SCA with cardiac origin may present different pathophysiology, clinical manifestations, and outcomes. The prognostic markers in cardiogenic arrest survivors may be different from those in all-causes SCA patients. Previous reports have indicated the phenomenon of the obesity paradox in SCA patients, but the results were not necessarily analogous to cardiogenic SCA patients. Opposite events or factors benefit outcomes during resuscitation or post-cardiac arrest care. Our current study indicated that obese patients had a higher risk of in-hospital mortality and unsatisfactory neurological recovery than the other patients. After adjusting for several crucial characteristics, CPR events, and post-arrest care, obesity remains associated with unsatisfactory prognosis. Furthermore, the subgroup analysis consistently showed that obesity adversely affected in-hospital mortality regardless of age, sex, pre-arrest comorbidities, CPR events, and post-arrest care (Fig. 3).

The current study had some limitations. First, because the study was retrospective in nature, selection bias could not be avoided. Whether the patients received emergency CAG was decided by the on-duty cardiologist. Also, the enrolment period was different in medical centers from 2011 through 2017 that caused potential selection bias. Second, the sample size of the current study was small. The low percentage of emergency CAG in this study may cause small sample size. Patients who both achieved sustained ROSC and were suspected cardiogenic arrest were indicated for emergency. The insignificance may be attributed from inadequate statistical power. Recruiting a large number of patients is necessary to increase the statistical power of the study and a propensity score matching would be further considered. Third, obesity type may be a crucial factor. Central obesity, characterized by excess fat accumulation in the abdominal area, differs from peripheral obesity in which excess fat accumulates in the adipose tissue of the buttocks, hips, and thighs^28^. A report indicated that people with central obesity had a higher mortality risk than those with similar BMI^29^. Forth, BMI is not the only index for evaluating obesity. Other parameters, such as waist circumference, waist-to-hip ratio, and body fat compartments, were reported to predict risk in patients with cardiac diseases^30^. BMI, a widely-accepted convenient measure for adiposity, does not adequately reflect obesity in some circumstances such as variation in body structures across age groups, sex, and ethnic group. Furthermore, BMI was measured upon index ICU admission. The effect of subsequent BMI change was not evaluated. Finally, since duration of CPR was longer in obese patients, quality of CPR can be an issue in obese patients, and may be better if CPR is done by EMS providers rather than bystanders. The data of EMS events including duration of hospital transportation and the time from call to hospital should be recorded in the future studies.

## Conclusions

In cardiogenic arrest survivors with significant CAD, obesity is associated with high in-hospital mortality and unsatisfactory neurological outcomes.

## Supplementary information

Supplementary Information.
