# Supplementary material for: Obese cardiogenic arrest survivors with significant coronary artery disease had worse in-hospital mortality and neurological outcomes
Source: Sci Rep. 2020 Oct 29;10:18638. doi: 10.1038/s41598-020-75752-9 (PMC7596497; doi:10.1038/s41598-020-75752-9)
Supplement: Supplementary file 1 — Supplementary Information. [file 41598_2020_75752_MOESM1_ESM.docx]

**Obese cardiogenic arrest survivors with significant coronary artery disease had worse in-hospital mortality and neurological outcomes**

Chih-Wei Sung, MD ^a^, Chien-Hua Huang, MD, PhD ^b^, Wen-Jone Chen, MD, PhD ^b,c^, Wei-Tien Chang, MD, PhD ^b^, Chih-Hung Wang, MD, PhD ^b^, Yen-Wen Wu, MD, PhD ^d^, Wei-Ting Chen, MD ^b^, Jia-How Chang, MD ^a^, Min-Shan Tsai, MD, PhD ^b^

^a^ Department of Emergency Medicine, National Taiwan University Hospital Hsin-Chu Branch, Hsinchu, Taiwan

^b^ Department of Emergency Medicine, National Taiwan University Medical College and Hospital, Taipei, Taiwan

^c^ Division of Cardiology, Department of Internal Medicine, National Taiwan University Hospital, Taipei, Taiwan

^d^ Department of Nuclear Medicine, Cardiology Division of Cardiovascular Medical Center, Far Eastern Memorial Hospital, New Taipei City, Taiwan

**Short title:** obesity in cardiogenic arrest with significant CAD

**Name and address of Corresponding authors:**

Min-Shan Tsai, M.D., Ph.D.

Department of Emergency Medicine, National Taiwan University Hospital

No.7, Chung-Shan S. Road, Taipei 100, Taiwan

Tel: 886-2-2356283

FAX: 886-2-23223150

E-mail: [mshanmshan@gmail.com](mailto:mshanmshan@gmail.com)

| **STable 1. The TIMI grade flow after PCI in each group** | | | | |
| --- | --- | --- | --- | --- |
| TIMI grade | underweight n = 6 | Normal-weight n = 52 | overweight n = 43 | obesity n = 21 |
| ≦1 | 0 (0%) | 1 (1.92%) | 4 (9.30%) | 3 (14.29%) |
| 2 | 3 (50.00%) | 13 (25.00%) | 18 (41.86%) | 8 (38.09%) |
| 3 | 3 (50.00%) | 38 (73.08%) | 21 (48.84%) | 10 (47.62%) |

TIMI = Thrombolysis in myocardial infarction; PCI= Percutaneous coronary intervention

Missing: 79 (39.3%)
